# Subacute Thyroiditis during the COVID-19 Pandemic: Searching for a Clinical Association with SARS-CoV-2

**DOI:** 10.1155/2021/5588592

**Published:** 2021-03-26

**Authors:** Pierpaolo Trimboli, Chiara Camponovo, Sebastiano Franscella, Enos Bernasconi, Niccolò Buetti

**Affiliations:** ^1^Clinic for Endocrinology and Diabetology, Lugano Regional Hospital, Ente Ospedaliero Cantonale, Lugano, Switzerland; ^2^Università della Svizzera Italiana (USI), Lugano, Switzerland; ^3^Division of Infectious Diseases, Lugano Regional Hospital, Ente Ospedaliero Cantonale, Lugano, Switzerland; ^4^Ente Ospedaliero Cantonale, Locarno Regional Hospital, Locarno, Switzerland; ^5^University of Paris, INSERM IAME, U1137, Team DeSCID, Paris, France; ^6^Infection Control Program and World Health Organization Collaborating Centre on Patient Safety, University Hospitals and Faculty of Medicine, University of Geneva, Geneva, Switzerland

## Abstract

**Purpose:**

To search for a clinical potential link between subacute thyroiditis (SAT) and severe acute respiratory syndrome coronavirus 2 (SARS-CoV-2) in a series of patients diagnosed with SAT during the COVID-19 pandemic, by retrospective evaluation of (1) clinical symptoms and (2) contact tracing.

**Methods:**

SAT  patients diagnosed from March to December 2020 were enrolled. The presence of typical clinical presentation of SARS-CoV-2, diagnostic tests for SARS-CoV-2, and contact with other individuals proven to be positive for SARS-CoV-2 were searched.

**Results:**

Ten SAT  cases were included. Fever was recorded in four patients. Cough, dyspnea, and headache were rarely reported. No patient had diagnosis of pneumonia. Two patients had moderate to severe fatigue after SAT. One patient experienced loss of smell and taste and had persistent fatigue over the following five months. No patient had positive SARS-CoV-2 diagnostic tests. At contact-tracing evaluation, only one patient had a contact with people who were diagnosed with SARS-CoV-2.

**Conclusions:**

Patients diagnosed with SAT during COVID-19 pandemic rarely experienced SARS-CoV-2-related symptomatology. The contact tracing did not show close contact with SARS-CoV-2 individuals in 9/10 cases.

## 1. Introduction

In March 2020, the World Health Organization declared the pandemic of novel coronavirus disease 2019 (COVID-19) caused by the severe acute respiratory syndrome coronavirus 2 (SARS-CoV-2) [1]. Since then, more than 70 million cases with more than 1.5 million deaths from SARS-CoV-2 were reported until December 2020 [2]. Coronaviruses are known to have a direct effect on endocrine glands and a direct damage by SARS-CoV to the thyroid follicular and parafollicular cells was demonstrated *in vitro* in 2007 [3]. During 2020, with the worldwide rapid diffusion of COVID-19, several data on the relationship between SARS-CoV-2 and thyroid disease have been emerging [4]. Some of these studies focused on the subacute thyroiditis (SAT), also known as De Quervain's thyroiditis, a self-limited inflammatory thyroid disease caused by several viruses and usually preceded by upper respiratory tract infections [5]. The clinical presentation of SAT encompasses neck pain, as the hallmark of the clinical syndrome, thyroid enlargement, general symptoms (e.g., mild fever and fatigue), and thyroid dysfunction. Classically, the clinical course of SAT includes three consecutive 2-month phases: (1) thyrotoxicosis due to the parenchymal damage; (2) hypothyroidism due to the deficiency of hormones during the initial restoring of parenchyma; (3) euthyroidism with the *restitutio ad integrum* of parenchyma [6]. Many viruses are known to be associated with the development of SAT, while a direct evidence of the presence of virus in the thyroid tissue is available only for a few cases [5]. In a recent full review of the literature about the impact of COVID-19 on the thyroid gland [4], the published cases of SAT during the COVID-19 pandemic were detailed. Although several clues could lead to a possible relationship between SAT and SARS-CoV-2, and the presence of the receptor for SARS-CoV-2 entry (i.e., angiotensin-converting enzyme 2 (ACE-2)) in the thyroid cells has been demonstrated [7]; a direct clinical association between SAT and SARS-CoV-2 has not been clarified.

The aim of the present study was to investigate the possible clinical relationship between SAT and SARS-CoV-2 in a series of patients diagnosed with SAT during the ongoing pandemic by retrospective (1) evaluation of the presence of typical symptoms of SARS-CoV-2 and (2) reconstruction of the history of contacts with other individuals diagnosed with SARS-CoV-2.

## 2. Methods

The study was conducted at the Clinic for Endocrinology and Diabetology of Lugano Regional Hospital located in the Italian-speaking region of Switzerland (Canton Ticino). In this region, two COVID-19 pandemic waves were observed, the first started in March and the second in October 2020.

All patients diagnosed with SAT during the period from March 1^st^ to December 15^th^, 2020, and recorded at our Clinic were enrolled in the study. The patients' clinical history was systematically searched for the presence of typical symptoms and signs of SARS-CoV-2 (i.e., fever, cough, dyspnea, headache, fatigue, pneumonia, loss of smell, and loss of taste) and they were classified according to onset's time of SAT (i.e., before, concomitant, or after). Specific reverse transcriptase polymerase chain reaction (rtPCR) in nasopharyngeal swabs and serum diagnostic tests for SARS-CoV-2 were recorded, when performed. The reconstruction of contact tracing of the included cases was conducted in December 2020 by a patients' interview to achieve information on their contact with other individuals proven to be positive for SARS-CoV-2 during the period between March to December 2020.

## 3. Results

The series included 10 patients (9 females and 1 male, mean age 47 years). The course of SAT was favorable in all cases with recovery. A low-dose (i.e., 20 mg per day) prednisone therapy was administered.

The most common symptoms of SARS-CoV-2 were searched and detailed in Table 1. Fever was recorded in five patients with no high body temperature and was concomitant with SAT. Cough, dyspnea, and headache were rarely experienced. No patient had diagnosis of pneumonia. Two patients had moderate to severe fatigue after SAT. One patient (#9) experienced loss of smell and taste after SAT and had persistent fatigue over the following five months. Four patients underwent SARS-CoV-2 nasopharyngeal swab while SARS-CoV-2 serum test was performed in two cases; no patient had positive SARS-CoV-2 diagnostic tests.

When we evaluated the contacts of our patients through standard contact-tracing interviews, we found that only one patient (#9) had a contact with a laboratory-confirmed SARS-CoV-2 subject and developed the typical COVID-19 symptomatology.

## 4. Discussion

The coronaviruses are known to be associated with the development of SAT, and an apparent increase of patients diagnosed with SAT during the COVID-19 pandemic has been reported [4]. However, to achieve the proof of a direct relationship between SARS-CoV-2 and SAT remains challenging [4]. To the best of our knowledge, here we reported the first series of SAT diagnosed during the COVID-19 pandemic in which both SARS-CoV-2-related symptoms and contact-tracing data were collected. Among our SAT patients, only one presented a typical clinical presentation of SARS-CoV-2 with persistence of fatigue over the following five months and had a close contact with a SARS-CoV-2-infected individual just before she developed SAT. However, she underwent diagnostic tests for SARS-CoV-2 (rtPCR in nasopharyngeal swab and serum) with negative results. In all the other patients, the symptomatology was not typical for SARS-CoV-2 and no positive SARS-CoV-2 tests were recorded. Overall, it was difficult in our series to demonstrate a clinical relationship between SAT  and SARS-CoV-2, while one case remained clinically suspicious for SARS-CoV-2 infection. To date, a small number of SAT patients with proven SARS-CoV-2 have been published [8–13]. All those cases had mild SARS-CoV-2 clinical presentation except one [10] and had a typical clinical course of SAT. According to the published cases, it seems that some patients with SARS-CoV-2 can be affected by SAT  and this should be taken into account also in those patients with mild SARS-CoV-2 manifestation. Interestingly, no patient of our series was placed in quarantine because of the lack of proof of SARS-CoV-2 infection, but nobody of their close contacts (i.e., family members, cohabitants, and colleagues) developed SARS-CoV-2-related symptoms. Therefore, SAT could not be considered as related to SARS-CoV-2 until proven otherwise.

SAT is presumed to be associated with viral infection or postviral inflammation. Indeed, a possible epidemiological linkage between enterovirus and thyroiditis has been described [5, 14]. Moreover, herpes viruses (e.g., cytomegalovirus and Epstein–Barr virus) have been considered in the pathogenesis of SAT [15–17]. However, it is an open issue whether they are responsible for thyroid diseases or there is only an epidemiological linkage based on seasonality or coincident increase of indirect markers (e.g., antibodies). It is reasonable that COVID-19 may influence the burden of SAT. SARS-CoV-2, probably due to its immunosuppressive effects in severe cases (i.e., lymphopenia [18]), may represent a risk factor for herpes viruses reactivation [19], thus leading to a subsequent thyroiditis. Moreover, it is possible that the inflammatory response produced by SARS-CoV-2 may influence further inflammation damages in thyroid follicles. All these pathogenic considerations, however, were speculative and our analysis appears to mitigate the probability of a clear association between SARS-CoV-2 infection and SAT. Further studies are needed to elucidate this association. For example, a case-control (i.e., including SAT patients and matched controls) study assessing serological response to COVID-19 may be a valuable option to unmask a possible association between SARS-CoV-2 and SAT. Such a study should be performed ideally before the broad introduction of SARS-CoV-2 vaccines. A direct cytopathic role of SARS-CoV-2, which appears to be unlikely, would necessitate the identification of viable virus in thyroid gland specimens.

Some specific considerations about our series of SAT have to be addressed. The present series was collected in a region (Canton Ticino, Southern Switzerland) which was particularly affected by COVID-19 pandemic with about 18,000 cases and 650 deaths until mid-December 2020 in a population of 350,000 inhabitants [20, 21]. In this Canton, a lockdown was implemented in the spring of 2020 (with complete stop of nonurgent thyroid visits in the regional hospitals) [22]. The patients were followed up at one single clinic and it was not possible to compare the frequency of SAT recorded during the study period with that of the previous year. A half of our patients did not undergo nasopharyngeal swab or serum test (see Table 1) and we do not have a solid proof in favor or against their SARS-CoV-2 infection. Most our cases were diagnosed during spring and summer, and a seasonal effect should be considered [14]. The possibility of false negative SARS-CoV-2 diagnostic tests, especially in asymptomatic patients, should be taken into account.

In conclusion, the present study showed that most patients diagnosed with SAT during COVID-19 pandemic rarely experienced the typical symptoms and signs of SARS-CoV-2. Indeed, according to contact tracing, only one patient met individuals diagnosed with SARS-CoV-2. These data do not exclude that SAT is an underestimated manifestation of SARS-CoV-2 and further studies are needed to clarify their possible association. Considering the rapid evolution of research on COVID-19, the present findings might be revised soon.

## Figures and Tables

**Table 1:** Clinical symptoms and contact-tracing data of the included patients.

	Age	Gender	Fever	Cough	Dyspnea	Headache	Fatigue	Pneumonia	Loss of smell	Loss of taste	SARS-CoV-2 nasopharyngeal swab	SARS-CoV-2 serum test	Contact with other individuals proved to be positive for SARS-CoV-2
1	36	F	—	—	—	—	c	—	—	—	—	—	—
2	53	F	—	—	—	—	a	—	—	—	—	b 2x neg	—
3	58	M	—	—	—	—	—	—	—	—	—	—	—
4	44	F	c (38.5)	—	—	—	—	—	—	—	c 1x neg	—	—
5	54	F	c (38.5)	—	b	c	—	—	—	—	—	—	—
6	44	F	c (38.5)	c	—	—	a	—	—	—	c 2x neg	—	—
7	66	F	—	—	—	—	c	—	—	—	—	—	—
8	46	F	—	—	—	—	—	—	—	—	—	—	—
9	39	F	c (38.0)	c	c		a	—	a	a	a 1x neg	b 2x neg	Yes, just before SAT
10	33	F	c (37.3)	—	—	—	—	—	—	—	b 2x neg	—	—

“a”, “b,” and “c” indicate if the symptom or feature was after, before, or concomitant to the SAT, respectively; the highest body temperature is indicated in parenthesis and is expressed in Celsius scale; “neg” means negative test; “2x” indicates repeated tests. The empty cells indicate the absence of the symptom or feature.

## Data Availability

The retrospective, blinded, grouped data used to support the findings of this study are available from the corresponding author upon request.

## Abbreviations

COVID-19: Coronavirus disease 2019
SARS-CoV-2: Severe acute respiratory syndrome coronavirus 2
SAT: Subacute thyroiditis.
